# Long-term body mass index changes in overweight and obese adults and the risk of heart failure, cardiovascular disease and mortality: a cohort study of over 260,000 adults in the UK

**DOI:** 10.1186/s12889-021-10606-1

**Published:** 2021-04-15

**Authors:** Barbara Iyen, Stephen Weng, Yana Vinogradova, Ralph K. Akyea, Nadeem Qureshi, Joe Kai

**Affiliations:** Primary Care Stratified Medicine group (PRISM), Division of Primary Care, University of Nottingham, Room 1402, Tower Building, Nottingham, NG7 2RD UK

**Keywords:** Obesity, Overweight, Body mass index (BMI), BMI trajectory, Cardiovascular disease, Heart failure

## Abstract

**Background:**

Although obesity is a well-recognised risk factor for cardiovascular disease (CVD), the impact of long-term body mass index (BMI) changes in overweight or obese adults, on the risk of heart failure, CVD and mortality has not been quantified.

**Methods:**

This population-based cohort study used routine UK primary care electronic health data linked to secondary care and death-registry records. We identified adults who were overweight or obese, free from CVD and who had repeated BMI measures. Using group-based trajectory modelling, we examined the BMI trajectories of these individuals and then determined incidence rates of CVD, heart failure and mortality associated with the different trajectories. Cox-proportional hazards regression determined hazards ratios for incident outcomes.

**Results:**

264,230 individuals (mean age 49.5 years (SD 12.7) and mean BMI 33.8 kg/m^2^ (SD 6.1)) were followed-up for a median duration of 10.9 years. Four BMI trajectories were identified, corresponding at baseline, with World Health Organisation BMI classifications for overweight, class-1, class-2 and class-3 obesity respectively. In all four groups, there was a small, stable upwards trajectory in BMI (mean BMI increase of 1.06 kg/m^2^ (± 3.8)). Compared with overweight individuals, class-3 obese individuals had hazards ratios (HR) of 3.26 (95% CI 2.98–3.57) for heart failure, HR of 2.72 (2.58–2.87) for all-cause mortality and HR of 3.31 (2.84–3.86) for CVD-related mortality, after adjusting for baseline demographic and cardiovascular risk factors.

**Conclusion:**

The majority of adults who are overweight or obese retain their degree of overweight or obesity over the long term. Individuals with stable severe obesity experience the worst heart failure, CVD and mortality outcomes. These findings highlight the high cardiovascular toll exacted by continuing failure to tackle obesity.

**Supplementary Information:**

The online version contains supplementary material available at 10.1186/s12889-021-10606-1.

## Background

Obesity and overweight increase the risk of cardiovascular disease [1] and heart failure [2], and are important causes of morbidity and mortality globally. In 2016, an estimated 1.9 billion adults globally were overweight, of which 650 million were obese [3]. In recent UK estimates from the Health Survey of England [4], 27% of adults were obese, and the proportion of overweight and obese adults increased from 53% in 1993 to 63% in 2017 [4].

A meta-analysis has shown that for every 5-unit increase in BMI, there is a 29% increase in the risk of coronary heart disease (CHD) which drops to 16% after adjustment for blood pressure and cholesterol levels [5]. Long-term weight loss in obese individuals through self-guided efforts or behavioural interventions is not always sustained. Most behavioural interventions in obese individuals achieve only modest weight loss ranging from only − 0.3 to − 3.1 kg at 12 months to − 0.3 to + 1.3 kg weight gain at 24 months [6], and many severely obese individuals remain severely obese for at least 5 years [7]. It is thought that a multi-disciplinary approach and intervention is required to prevent obesity, reduce progression to more severe forms and lead to substantive and sustainable public health outcomes [8]. While weight loss interventions may improve cardiovascular risk factors [9], metabolic function [10] or even reverse type 2 diabetes [11], it is unclear what the long term effect of weight changes are on cardiovascular endpoints.

Few studies have explored how BMI in overweight or obese individuals may change over time, and any cardiovascular impact this may have. No existing research on BMI trajectories over time [12–16] has focused entirely on obese and overweight adult populations, so the BMI course of these individuals is unknown. This aim of this study was firstly to examine BMI trajectories in the general population of adults who were overweight or obese adults; and secondly to explore the risk of heart failure, cardiovascular disease (CVD), CVD-related mortality and all-cause mortality, associated with different BMI trajectories.

## Methods

### Source of data

We conducted a cohort study using data from the UK Clinical Practice Research Datalink (CPRD), a nationally representative database of routinely recorded primary care electronic health records [17]. General practice is the first point of contact for non-emergency healthcare in the UK, and over 98% of the UK population are registered with a General Practitioner. The CPRD database contains anonymised longitudinal data entered during routine consultations from general practices who have agreed to provide patient data. The database has a coverage rate of approximately 15% of the UK population and patients are broadly representative of the UK general population in terms of age, sex and ethnicity [17]. Patient records were available from 790 general practices which contributed to the database during the study period from 1999 to 2018. Primary care records from CPRD were linked with secondary care records (Hospital Episode Statistics) and death registration records from the Office of National Statistics (ONS). Data access and ethical approval was granted by the CPRD Independent Scientific Advisory Committee (Protocol number 18_195) in August 2018.

### Study participants and covariates

We identified individuals aged 18 years or older, with a recorded or computed BMI (weight divided by the square of height) of 25 kg/m^2^ or greater, and subsequent records of BMI during the study period. Each individual could have up to five BMI data points – the first recorded overweight or obese BMI was defined as the baseline BMI, and then subsequent BMI measures at 2 years (±90 days), 5 years (±6 months), 8 years (±6 months) and 10 years (±12 months). To be included in the study, participants had to be registered with the general practice for at least one year before the date of their baseline BMI. Individuals with pre-existing records of CVD (defined as coronary heart disease, peripheral vascular disease, stroke or transient ischaemic attack) or heart failure, were excluded from the study. All participants were followed up until diagnosis of CVD, heart failure, death, transfer out of the practice or last date of data collection, whichever occurred first. To ensure that there were sufficient BMI records for the trajectory analyses, we included only individuals who had BMI records at baseline and at least one other defined time point, prior to the incidence of CVD, heart failure or death.

In all study participants, we collected data on covariates such as patient demographics (age, sex, ethnicity and socioeconomic status) as well as comorbidities that could alter their risk of developing CVD or heart failure [18]. Our measure of material socioeconomic deprivation was the 2015 English index of multiple deprivation (IMD) in quintiles [19], and this was available for individuals with linked secondary-care records. Records of comorbidities collected at baseline were type-2 diabetes, atrial fibrillation, chronic kidney disease, hypertension, rheumatoid arthritis and other inflammatory diseases. Smoking status and records of alcohol consumption were collected at baseline as well as at the 2-year, 5-year, 8-year and 10-year follow-up time points.

### Outcome ascertainment

Incident CVD was defined as any new clinical diagnosis of coronary heart disease, stroke, transient ischaemic attack (TIA) or peripheral vascular disease. CVD, heart failure and death records were identified from individuals’ primary care, secondary care and ONS death registry records during the study period. Disease codes used for identification of CVD and heart failure are shown in the supplementary online file.

### Analyses

Analyses were conducted using Stata SE version 15. Baseline descriptive statistics were presented for the entire study population, including missing data. Although the inclusion criteria ensured that individuals had a minimum of 2 BMI records (BMI at baseline and a minimum of one other BMI record), all values of BMI that were missing at the study set time points and before the incidence of CVD, heart failure or death, were estimated using multiple imputation by chained equations procedure. This approach provides estimates for missing values when data are assumed to be missing at random [20]. It is also the recommended approach for handing missing weight data in epidemiological studies using primary care health records [21], where the practical analytical approach is to include in the imputation model, variables that are predictive of the missing data [22]. Previous research has shown that the use of multiple imputation for missing weight records in primary care databases provided results comparable with population surveys [23]. Baseline BMI, demographic variables such as age and sex, clinical comorbidities (cardiovascular disease, diabetes, hypertension, rheumatoid arthritis and other inflammatory conditions, chronic kidney disease and atrial fibrillation), smoking status and alcohol consumption were included in the imputation models to create 10 imputed datasets. Body mass index measures at baseline, 2 years, 5 years, 8 years and 10 years were used to assign individuals into trajectories of BMI using group based trajectory modelling (GBTM). GBTM provides a statistical method to identify distinctive clusters of individuals who follow a similar developmental trajectory and enables profiling of the characteristics of individuals within the clusters [24]. In the GBTM models, BMI was the dependent variable and time (baseline, 2, 5, 8 and 10 year time-points) was the independent variable. Models took account of the effect of time-varying covariates: age, smoking status and alcohol consumption on individuals’ probability of group membership, and assigned individuals to the group to which they had the highest probability. The Stata plug-in program (*Traj*) was used to estimate group-based trajectory models using the maximum likelihood estimation method [25]. Data was modelled using the censored normal distribution and BMI values that were considered clinically implausible (BMI less than 10 kg/m^2^ or greater than 131 kg/m^2^) were excluded from analyses. The Bayesian information criterion (BIC) was used as criterion for selection of the best-fitting trajectory model whereby models with the lower value of BIC was preferred. BIC captures generalised trends over time while also minimising the risk of over-fitting the models. While the Akaike’s information criterion (AIC) and BIC both aim at achieving a compromise between model goodness of fit and model complexity, with maximum likelihood estimates driven to penalise free parameters, BIC are more stringent than AIC [26] and is asymptotically consistent, in that it will select the true model if, among other assumptions, the true model is among the candidate models considered [27]. Similar to GBTM methods employed in a previous study [28], we identified the ideal number of trajectory groups for our study population, by estimating the BIC in 2- group models, 3-group models and 4-group models. This was followed by testing zero-order, linear, quadratic and cubic specifications for the different trajectory shapes until the best fitting shape was derived. In selecting the final model, we ensured each trajectory group had a minimum of 5% of the study population. The 4-group model with four cubic trajectories was selected as the model with the best-fit due to the low BIC value as well as having an adequate proportion of the study population per trajectory group (BIC data are shown in supplementary Table 1).

Socio-demographic characteristics, clinical profile, comorbidities as well as CVD, heart failure and mortality outcomes were assessed for individuals in the trajectory groups. Baseline characteristics of individuals between BMI trajectory groups were compared using the ANOVA test for continuous variables or the chi-test for categorical variables. We used survival analyses to estimate the incidence rates of outcomes for individuals in the different trajectory groups. The proportional hazards assumption was checked using statistical tests (Schoenfeld residuals). Multivariate Cox proportional hazards modelling was used to derive hazards ratios for CVD, heart failure and mortality in the groups, adjusting for demographic and clinical covariates that were significantly associated with the exposure and outcome. The lowest BMI trajectory group, the overweight trajectory group, was the reference for comparison because it was the largest and most normative group. We then estimated the mean change in BMI over the 10 year period, in the subgroup of individuals who had BMI data at 10 years. To assess the validity of our assumption that missing data were missing at random, a sensitivity analyses was done using complete case analyses. Owing to the possibility that not all CVD events are captured are recorded in primary care records, further sensitivity analyses was restricted to only individuals whose primary care records were linked with secondary care (hospital episode statistics) and the ONS (for mortality records).

## Results

### Study population

**Fig. 1:**
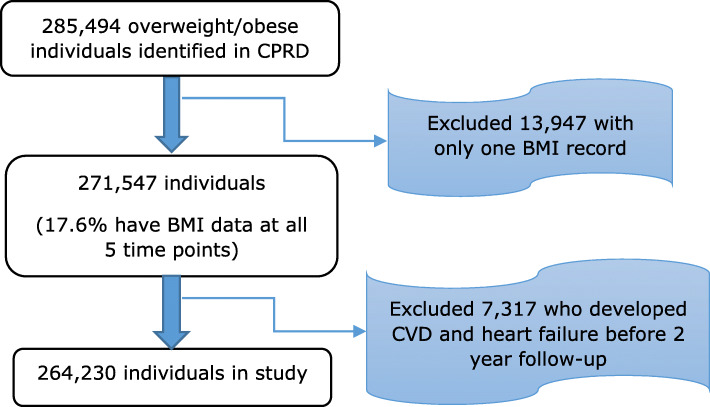
Flow chart showing how the study population of overweight and obese subjects were derived

A total of 264,230 overweight and obese individuals were included in the study. The flowchart in Fig. 1 demonstrates how the study population were derived from the overall CPRD population of obese and overweight subjects.

Baseline characteristics of the study subjects are shown in Table 1. Females comprised 62% of individuals in the study, the mean age at baseline was 42.5 (SD 12.7) years and the mean BMI was 33.8 (SD 6.1) kg/m^2^. Linkage with hospital and death registration data was available for 138,755 (52.5%) individuals in CPRD. There were complete BMI records at all 5 set time points for 17.3% of the study population. 20% of subjects had 4 BMI records and 30% had 3 BMI records. Multiple imputation was used to estimate missing BMI values to ensure BMI records were available at all set times prior to the incidence of CVD, heart failure or death. Ethnicity records were available for 69.2% of the study population, with the majority of individuals being white (63.7%). The most prevalent comorbidities at baseline were hypertension (19.9%) and type 2 diabetes (8.6%).

**Table 1:** Baseline characteristics of the study population

	Total n (%) *N* = 264,230	Male n (%) *N* = 99,590	Female n (%) *N* = 164,640
**Age in years (mean (SD))**	49.5 (12.7)	51.2 (11.7)	48.5 (13.2)
**Body mass index (kg/m**^**2**^**)**	33.8 (6.1)	33.2 (5.4)	34.2 (6.4)
**Ethnicity**			
White	168,330 (63.7)	62,873 (63.1)	105,457 (64.1)
Black African	2154 (0.8)	548 (0.6)	1606 (1.0)
Black Caribbean	2059 (0.8)	495 (0.5)	1564 (1.0)
Black other	595 (0.2)	146 (0.2)	449 (0.3)
Bangladeshi	334 (0.1)	98 (0.1)	236 (0.1)
Chinese	158 (0.1)	56 (0.1)	102 (0.1)
Indian	2908 (1.1)	1032 (1.0)	1876 (1.1)
Pakistani	1852 (0.7)	603 (0.6)	1249 (0.8)
Mixed	595 (0.2)	170 (0.2)	425 (0.3)
Other Asian	1306 (0.5)	518 (0.5)	788 (0.5)
Other (unspecified)	2498 (1.0)	920 (0.9)	1578 (1.0)
Missing ethnicity records	81,441 (30.8)	32,131 (32.3)	49,310 (30.0)
**IMD quintile**			
1 (least deprived)	25,534 (9.7)	10,186 (10.2)	15,348 (9.3)
2	29,958 (11.3)	11,404 (11.5)	18,554 (11.3)
3	28,070 (10.6)	10,260 (10.3)	17,810 (10.8)
4	30,167 (11.4)	10,697(10.7)	19,470 (11.8)
5 (most deprived)	24,911 (9.4)	8517 (8.6)	16,394 (10.0)
Missing records	125,590 (47.5)	48,526 (48.7)	77,064 (46.8)
**Smoking status**			
Ex-smoker	78,539 (29.7)	37,187 (37.3)	41,352 (25.1)
Light smoker (1–9 cigs/day)	18,053 (6.8)	7209 (7.2)	10,844 (6.6)
Moderate smoker (10–19/day)	13,182 (5.0)	4173 (4.2)	9009 (5.5)
Heavy smoker (20–39/day)	11,213 (4.2)	4622 (4.6)	6591 (4.0)
Non-smoker	142,969 (54.1)	46,289 (46.5)	96,680 (58.7)
Missing	274 (0.1)	110 (0.1)	164 (0.1)
**Alcohol consumption**			
Trivial (< 1 unit/day)	88,408 (33.5)	28,902 (29.0)	59,506 (36.1)
Light (1–2 units/day)	39,423 (14.9)	19,986 (20.1)	19,437 (11.8)
Moderate (3–6 units/day)	22,394 (8.5)	13,231 (13.3)	9163 (5.6)
Heavy (7–9 units/day)	7090 (2.7)	5271 (5.3)	1819 (1.1)
Very heavy (> 9 units/day)	4377 (1.7)	3621 (3.6)	756 (0.5)
Ex-alcohol consumption	9660 (3.7)	4477 (4.5)	5183 (3.2)
No alcohol consumption	84,698 (32.1)	21,433 (21.5)	63,265 (38.4)
Missing	8180 (3.1)	2669 (2.7)	5511 (3.4)
**Co-morbidities**			
Atrial fibrillation	2144 (0.8)	1266 (1.3)	878 (0.5)
Chronic kidney disease	2460 (0.9)	953 (1.0)	1507 (0.9)
Hypertension	52,574 (19.9)	22,203 (22.3)	30,371 (18.4)
Type-2 diabetes	22,844 (8.6)	11,682 (11.7)	11,162 (6.8)
Rheumatoid arthritis /inflammatory diseases	4857 (1.8)	1078 (1.1)	3779 (2.3)

In the study population of 264,230 overweight or obese adults, we identified four distinct BMI trajectories over time, with a statistically significant difference in the BMI at baseline for individuals in the different trajectories (*p* < 0.001). The estimates for posterior probability of group membership are shown in Supplementary Table 2. Across the trajectory groups, the average posterior probability was greater than 0.93, which is above the recommended minimum average probability of 0.70, and the odds of correct classification for the BMI GBTM groups were above 30 for all groups, indicating good accuracy of model assignment (Supplementary Table 2).

**Fig. 2:**
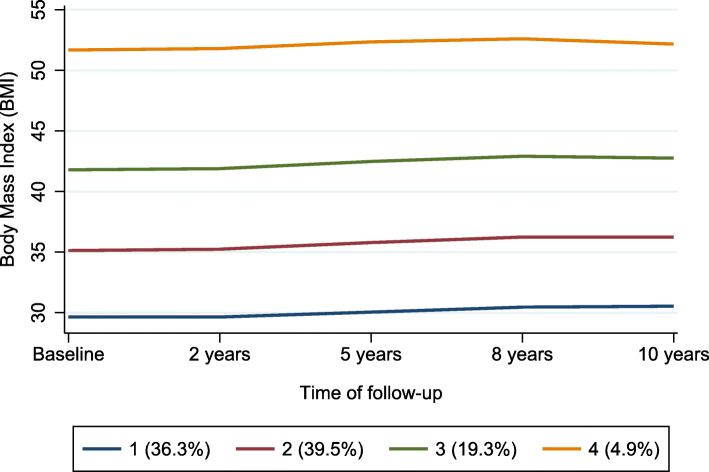
Body mass index (BMI) trajectories using BMI measures at baseline and then follow-up at 2 years, 5 years, 8 years and 10 years. Percentages below plot represent percentage of study population within each trajectory group. Mean BMI change in trajectory group 1 (overweight-stable group): + 0.99 (SD 3.10) kg/m^2 .^ Mean BMI change in trajectory group 2 (obese class 1-stable group): + 1.19 (SD 1.67) kg/m^2^. Mean BMI change in trajectory group 3 (obese class 2-stable group): + 1.04 (SD 4.59) kg/m^2^ Mean BMI change in trajectory group 4 (obese class 3-stable group): + 0.62 (SD 6.27) kg/m^2^

The mean BMI at baseline, for individuals in trajectory group 1 (*n* = 95,944, 36.3%) was 28.7 kg/m^2^, corresponding to the WHO overweight BMI category. Individuals in trajectory group 2 (*n* = 104,616, 39.6%) had a mean baseline BMI of 33.7 kg/m^2^ corresponding to WHO class 1 obesity category. The mean baseline BMI in trajectory group 3 (*n* = 50,866, 19.3%) was 39.9 kg/m^2^ corresponding to WHO class 2 obesity while those in trajectory group 4 (*n* = 12,804, 4.9%) had a mean baseline BMI of 49.1 kg/m^2^ corresponding to WHO class 3 obesity. Although BMI remained relatively stable across the 4 trajectory groups, individuals had a mean BMI increase of 1.06 kg/m^2^ (± 3.8) over 10 years (Fig. 2).

The characteristics of individuals belonging to the different BMI trajectory groups are shown in Table 2. Compared to other trajectory groups, the obese class 3-stable trajectory group comprised of the highest proportion of females. There were greater levels of deprivation among particularly the most severely obese trajectory groups. There was also an increasing trend in the prevalence of clinical morbidities such as hypertension, atrial fibrillation, chronic kidney disease and type 2 diabetes, with increasing severity of obesity, such that individuals in the overweight-stable trajectory group had the lowest prevalence whereas those in obese class 3-stable trajectory group had the highest prevalence of comorbidities at baseline.

**Table 2:** Sociodemographic and clinical characteristics by BMI trajectory groups (N = 264,230)

	Trajectory 1 (overweight-S) (n = 95,944)	Trajectory 2 (Obese 1-S) (n = 104,616)	Trajectory 3 (obese 2-S) (n = 50,866)	Trajectory 4 (obese 3-S) (n = 12,804)
**Patient characteristics (Sociodemographic characteristics)**				
**Age at start of study (mean (SD))**	49.5 (13.2)	49.9 (12.7)	49.1 (12.2)	48.8 (11.2)
**Male gender n (%)**	37,882 (39.5)	41,685 (39.9)	16,652 (32.7)	3371 (26.3)
**Smoking**				
Ex-smoker	27,950 (29.1)	31,763 (30.4)	15,123 (29.7)	3703 (28.9)
Light smoker (1–9 cigs/day)	6918 (7.2)	7071 (6.8)	3270 (6.4)	794 (6.2)
Moderate smoker (10–19 cigs/day)	5118 (5.3)	5214 (5.0)	2347 (4.6)	503 (3.9)
Heavy smoker (20–39 cigs/day)	3827 (4.0)	4470 (4.3)	2297 (4.5)	619 (4.8)
Non-smoker	52,070 (54.3)	55,989 (53.5)	27,757 (54.6)	7153 (55.9)
Missing	61 (0.1)	109 (0.1)	72 (0.1)	32 (0.3)
**Alcohol consumption**				
Trivial (< 1 unit/day)	31,978 (33.3)	35,035 (33.5)	17,192 (33.8)	4203 (32.8)
Light (1–2 units/day)	16,405 (17.1)	15,773 (15.1)	6133 (12.1)	1112 (8.7)
Moderate (3–6 units/day)	9746 (10.2)	8748 (8.4)	3314 (6.5)	586 (4.6)
Heavy (7–9 units/day)	2883 (3.0)	2961 (2.8)	1076 (2.1)	170 (1.3)
Very heavy (> 9 units/day)	1621 (1.7)	1905 (1.8)	722 (1.4)	129 (1.0)
Ex-alcohol consumption	3086 (3.2)	3759 (3.6)	2174 (4.3)	641 (5.0)
No alcohol consumption	28,076 (29.3)	33,168 (31.7)	18,113 (35.6)	5341 (41.7)
Missing	2149 (2.2)	3267 (3.1)	2142 (4.2)	622 (4.9)
**Deprivation (IMD quintile)**				
1 (least deprived)	10,122 (10.6)	10,251 (9.8)	4334 (8.5)	827 (6.5)
2	11,043 (11.5)	12,124 (11.6)	5549 (10.9)	1242 (9.7)
3	9644 (10.1)	11,386 (10.9)	5654 (11.1)	1386 (10.8)
4	9889 (10.3)	12,173 (11.6)	6412 (12.6)	1693 (13.2)
5 (most deprived)	7528 (7.9)	9818 (9.4)	5799 (11.4)	1766 (13.8)
Missing records	47,718 (49.7)	48,864 (46.7)	23,118 (45.5)	5890 (46.0)
**Weight profile and clinical characteristics**				
**Body mass index in kg/m**^**2**^ **(mean (SD))**				
Baseline BMI	28.7 (2.2)	33.7 (2.7)	39.9 (3.5)	49.1 (5.7)
BMI at 2 years	28.6 (2.2)	33.7 (2.4)	39.9 (3.2)	49.1 (5.4)
BMI at 5 years	29.2 (2.3)	34.5 (2.4)	40.8 (3.1)	50.0 (5.3)
BMI at 8 years	29.5 (2.5)	34.8 (2.6)	41.1 (3.3)	50.1 (5.4)
BMI at 10 years	29.7 (2.8)	34.9 (3.0)	41.1 (3.7)	49.8 (5.7)
Mean BMI change over 10 years	1.0 (3.1)	1.2 (3.7)	1.0 (4.6)	0.6 (6.3)
**Prevalence of comorbidities at baseline**				
Atrial fibrillation	671 (0.7)	865 (0.8)	452 (0.9)	156 (1.2)
Chronic kidney disease	771 (0.8)	998 (1.0)	532 (1.0)	159 (1.2)
Hypertension	15,708 (16.4)	21,438 (20.5)	12,009 (23.6)	3419 (26.7)
Type-2 diabetes	6384 (6.7)	8930 (8.5)	5609 (11.0)	1921 (15.0)
Rheum arthritis/inflammatory diseases	1674 (1.7)	1970 (1.9)	952 (1.9)	261 (2.0)

### Cardiovascular disease, heart failure and mortality outcomes

There were a total of 30,400 incident cases of cardiovascular disease over 2,829,075 person-years of follow-up (median follow-up of 10.9 years (IQR 7.0–14.1)). Table 3 shows the overall CVD incidence rates as well as incidence rates of coronary heart disease (CHD), stroke/transient ischemic attack (TIA), peripheral vascular disease, heart failure, and mortality in the trajectory groups. The incidence rate of CVD in the entire study population (per 1000 person-years) was 10.75 (95% CI 10.61–10.87). The CVD incidence rate (per 1000 person-years) among individuals in the overweight-stable trajectory group was 9.30 (9.12–9.49). Higher incidence rates of overall CVD and CVD subtypes were observed in obese class 1-stable trajectory group compared to overweight-stable individuals but no further increase in incidence rates of CHD, stroke/TIA or peripheral vascular disease, with more severe categories of obesity from obese class 1-stable to obese class 3-stable groups. There was however a substantial and significant gradient in heart failure incidence with increasing severity of obesity from overweight-stable to obese class 3-stable trajectory groups, such that heart failure incidence rate (per 1000 person years) in overweight-stable trajectory individuals was 1.70 (1.6–1.8), and 5.70 (5.3–6.1) in obese class 3-stable trajectory individuals (score test for trend across BMI groups *p* < 0.0001).

**Table 3:** Cardiovascular disease, heart failure and death in the BMI trajectory groups

	Trajectory 1 (overweight-S) (n = 95,944)	Trajectory 2 (obese class 1-S) (n = 104,616)	Trajectory 3 (obese class 2-S) (n = 50,866)	Trajectory 4 (obese class 3-S) (n = 12,804)
***Cardiovascular disease and heart failure outcomes***				
Age (years) at first CVD diagnosis (mean (SD))	65.9 (12.0)	66.2 (11.8)	65.2 (11.8)	64.1 (10.9)
Follow-up (1000 person years)	1100	1100	517.7	123.1
Number of events (%)				
Any CVD	10,054 (10.5)	12,666 (12.1)	6137 (12.1)	1543 (12.1)
CHD	6074 (6.3)	7569 (7.2)	3329 (6.5)	714 (5.6)
Stroke/TIA	2675 (2.8)	3152 (3.0)	1436 (2.8)	300 (2.3)
PVD	1246 (1.3)	1425 (1.4)	595 (1.2)	108 (0.8)
Heart failure	1836 (1.9)	3175 (3.0)	1947 (3.8)	702 (5.5)
***Incidence rate (95% CI) of CVD and heart failure, per (1000 person-years)***				
Any CVD	9.30 (9.1–9.5)	11.44 (11.2–11.6)	11.86 (11.6–12.2)	12.54 (11.9–13.2)
Coronary heart disease	5.62 (5.5–5.8)	6.83 (6.7–7.0)	6.43 (6.2–6.7)	5.80 (5.4–6.2)
Stroke/TIA	2.48 (2.4–2.6)	2.85 (2.7–2.90)	2.77 (2.6–2.9)	2.44 (2.2–2.7)
Peripheral vascular disease	1.15 (1.1–1.2)	1.29 (1.2–1.4)	1.15 (1.1–1.2)	0.88 (0.7–1.1)
Heart failure	1.70 (1.6–1.8)	2.87 (2.8–3.0)	3.76 (3.6–3.9)	5.70 (5.3–6.1)
***Mortality outcomes***				
Deaths during follow-up n(%) (total *n* = 24,022)	7938 (8.3)	9175 (8.8)	5092 (10.0)	1817 (14.2)
Age (years) at death (mean (SD))	74.5 (12.1)	73.6 (12.0)	70.9 (12.3)	67.3 (11.8)
CVD related death n(%) (total *n* = 2827)	761 (0.8)	1165 (1.1)	679 (1.3)	222 (1.7)
***Mortality rate (95% CI), number of events per 1000 person-years***				
All-cause mortality rate	7.35 (7.2–7.5)	8.28 (8.1–8.5)	9.84 (9.6–10.1)	14.76 (14.1–15.5)
CVD-related mortality rate	0.70 (0.7–0.8)	1.05 (1.0–1.1)	1.31 (1.2–1.4)	1.80 (1.6–2.1)

A total of 24,022 deaths occurred during the period of follow-up, of which 2827 (11.8%) were cardiovascular deaths. The overall mortality rate in the study population (per 1000 person-years) was 8.5 (8.4–8.6). All-cause mortality and CVD-related mortality rates increased with more severe categories of obesity (shown in Table 3). As well as having the earliest onset of incident CVD, obese class 3-stable trajectory individuals had the highest all-cause mortality rate, CVD-related mortality rate and the youngest age at death.

Table 4 shows the hazards ratios for CVD, heart failure and mortality outcomes for individuals in obese 1-stable, obese 2-stable and obese 3-stable trajectory groups, compared to individuals in the overweight-stable trajectory group. After adjusting for age, sex and comorbidities, individuals in obese class 1, 2 and 3 trajectory groups had higher risks of all CVD, heart failure and mortality outcomes, compared to individuals in the overweight-stable trajectory group. An increase in coronary heart disease (HR 1.14 (95% CI 1.10–1.18)) and stroke/TIA risk (HR 1.09 (1.03–1.15)) was observed in obese class 1-stable compared to overweight individuals but no further increase in risk of these conditions were found with more severe categories of obesity. In the most severely obese (obese 3-stable) group, there was no statistically significant difference in the risk of coronary heart disease (HR 1.06 (0.99–1.16)) and stroke (HR 1.04 (0.92–1.18)), compared to individuals in the overweight-stable group. The risk of peripheral vascular disease did not differ significantly between those in the overweight, obese 1-stable and obese 2-stable trajectory groups. There was however, a reduced risk of peripheral vascular disease risk in obese 3-stable adults compared to those in the overweight-stable group (HR 0.73 (0.60–0.89)).

**Table 4:** Risk of CVD outcomes and mortality associated with BMI trajectory groups

		Unadjusted Hazards ratio (95% CI)	Adjusted hazards ratio (95% CI)	
CVD outcome	Trajectory group		Model adjusted for age and sex	Model adjusted for age, sex and comorbidities^a^
**Overall CVD**	overweight-S	1.00	1.00	1.00
	obese 1-S	1.25 (1.22–1.28)	1.24 (1.21–1.28)	1.15 (1.12–1.19)
	obese 2-S	1.32 (1.28–1.36)	1.46 (1.41–1.51)	1.27 (1.23–1.31)
	obese 3-S	1.42 (1.35–1.50)	1.75 (1.66–1.85)	1.40 (1.33–1.48
**Coronary heart disease**	overweight-S	1.00	1.00	1.00
	obese 1-S	1.23 (1.19–1.28)	1.23 (1.18–1.27)	1.14 (1.10–1.18)
	obese 2-S	1.17 (1.12–1.22)	1.30 (1.24–1.35)	1.13 (1.09–1.18)
	obese 3-S	1.07 (0.99–1.16)	1.31 (1.22–1.42)	1.06 (0.99–1.16)
**Stroke/ TIA**	overweight-S	1.00	1.00	1.00
	obese 1-S	1.18 (1.12–1.24)	1.16 (1.10–1.22)	1.09 (1.03–1.15)
	obese 2-S	1.16 (1.09–1.24)	1.27 (1.19–1.36)	1.13 (1.06–1.20)
	obese 3-S	1.04 (0.93–1.18)	1.26 (1.12–1.42)	1.04 (0.92–1.18)
**Peripheral vascular disease**	overweight-S	1.00	1.00	1.00
	obese 1-S	1.13 (1.05–1.22)	1.13 (1.04–1.22)	0.99 (0.92–1.07)
	obese 2-S	1.02 (0.92–1.12)	1.16 (1.06–1.29)	0.92 (0.83–1.01)
	obese 3-S	0.79 (0.65–0.96)	1.03 (0.85–1.25)	0.73 (0.60–0.89)
**Heart failure**	overweight-S	1.00	1.00	1.00
	obese 1-S	1.73 (1.63–1.83)	1.73 (1.63–1.83)	1.51 (1.42–1.60)
	obese 2-S	2.31 (2.16–2.46)	2.70 (2.53–2.88)	2.09 (1.96–2.23)
	obese 3-S	3.58 (3.28–3.91)	4.94 (4.53–5.40)	3.26 (2.98–3.57)
**All-cause mortality**	overweight-S	1.00	1.00	1.00
	obese 1-S	1.16 (1.13–1.20)	1.16 (1.12–1.19)	1.12 (1.09–1.16)
	obese 2-S	1.41 (1.36–1.46)	1.63 (1.58–1.69)	1.55 (1.50–1.61)
	obese 3-S	2.17 (2.06–2.29)	2.96 (2.81–3.12)	2.72 (2.58–2.87)
**CVD-related deaths**	overweight-S	1.00	1.00	1.00
	obese 1-S	1.53 (1.40–1.68)	1.54 (1.40–1.68)	1.44 (1.31–1.58)
	obese 2-S	1.94 (1.75–2.15)	2.33 (2.10–2.59)	2.06 (1.86–2.29)
	obese 3-S	2.73 (2.35–3.17)	3.98 (3.42–4.63)	3.31 (2.84–3.86)

Trajectory group 1 (overweight-stable) used as baseline group for comparison

^a^ Multivariate cox regression models adjusted for age, sex, hypertension, type 2 diabetes, atrial fibrillation and chronic kidney disease

The risk of heart failure, all-cause mortality and CVD-related mortality increased considerably with increasing severity of obesity such that after adjusting for age, sex and comorbidities, individuals in obese class 3-stable trajectory group had hazards ratios of 3.3 for heart failure, 3.3 for CVD-related deaths and 2.7-for all-cause mortality, compared to individuals in the overweight-stable trajectory group.

**Fig. 3:**
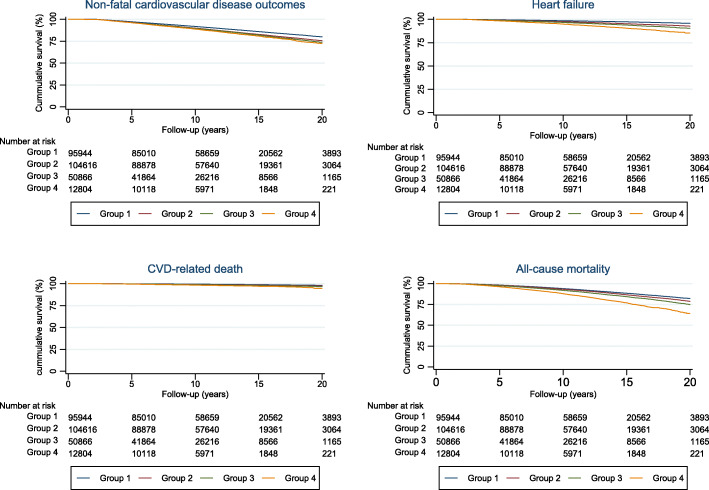
Kaplan Meier survival plots showing cumulative outcome-free survival of individuals in the 4 BMI trajectory groups. With increasing BMI trajectory groups, individuals had higher risks of non-fatal cardiovascular outcomes, heart failure and mortality

Figure 3 illustrates the cumulative outcome-free survival of individuals in the trajectory groups.

In sensitivity analyses done using only the BMI records available in patients’ electronic health records (without multiple imputation of missing BMI records), four BMI trajectory groups were identified and the BMI measures across the BMI trajectories were similar to measures in the main study which used multiple imputation to estimate missing BMI records. Also, similar to findings in the main study, there was a small stable upwards trajectory across all 4 groups, with an overall mean BMI increase of 1.26 kg/m2 (SD 4.47) over 10 years (supplementary Table 3 and supplementary Figure 1).

In further sensitivity analyses restricted to 138,755 individuals whose primary care records were linked with secondary care and ONS death registration records, the hazards ratios for overall CVD, coronary heart disease, stroke/TIA, heart failure, all-cause mortality and CVD-related death remained significantly higher in obese class 1-stable, class 2-stable and class 3-stable individuals, compared with the overweight-stable trajectory group of individuals. As in the main analyses, there was no observed increase in the risk of peripheral vascular disease in adults who were obese compared to overweight adults (supplementary Table 4).

## Discussion

### Main findings of paper

In this large general population cohort study of adults who were overweight or obese, we observed a stable upwards BMI trajectory over time whereby most subjects retained their degree of obesity over the long term. The overall risk of CVD, heart failure and mortality increased with increasing severity of obesity. Whilst there was no significant increase in risk of coronary heart disease and stroke in the most severely obese group, the increase in CVD risk was most marked for heart failure and mortality. After adjusting for the effect of age, sex and comorbidities, individuals in the most severely obese group had a 3.3-fold higher risk of heart failure, 3.3 fold higher risk of CVD-related mortality and 2.7-fold higher risk of all-cause mortality compared with overweight individuals. There were greater levels of socioeconomic deprivation with increasing severity of obesity, confirming that this is disproportionately an issue in the materially deprived.

### Strengths and limitations

To our knowledge, this is the first and largest study to analyse overweight and obese adults’ BMI trajectories and their impact on CVD endpoints, heart failure, and mortality. We had a large sample size of obese and overweight individuals who were studied prospectively with multiple BMI measures per individual over an extensive follow-up period. Linkage of individuals’ routine electronic primary care records to their secondary care and death registration records, enabled more robust extrapolation of CVD diagnosis and mortality data. The use of health professional-recorded rather than self-reported BMI measures minimised the risk of inaccuracies in the study. By using data from a large nationally representative database of UK electronic health records, the study findings can be generalised to the general population of overweight and obese adults.

Some limitations of this study are recognised. Body mass index is a surrogate measure of adiposity. Body composition of fat and skeletal muscle mass changes with age [29] and differs between sexes and ethnic groups [30]. While other indices such as waist-hip ratio and waist circumference are more suitable and accurate measures of adiposity than BMI, these are not used routinely in clinical practice and are not routinely available in electronic heath records. Over 60% of our study population were White, and so the CVD risk profile and CVD-related outcomes in the study population may not be directly generalizable to, or may underestimate obesity-related heart failure, CVD and mortality risk in other ethnic populations. There was no information on physical activity level or dietary intake so it remains unclear whether weight change observed in individuals’ was intentional or non-intentional and due to presence of disease. Lastly, a study inclusion criterion was a minimum of 2 BMI entries in subjects’ primary care records so there is a small risk of selection bias in the population studied. We had missing BMI data and acknowledge that this can constitute considerable challenges in the analyses and interpretation of results as well as potentially weaken the validity of results and conclusion [20]. However, we estimated these missing BMI records using multiple imputation based on the missing at random assumption. A sensitivity analysis examined the effect of this assumption and found that results of analyses using only available BMI records were similar to results of analyses using multiple imputation.

### Comparison with existing literature

This is the first study to evaluate the long-term impact of overweight and obese individuals’ BMI trajectory on cardiovascular endpoints, heart failure and mortality outcomes. While the association between obesity and cardiovascular disease is established [1, 2], our study sought to assess the effect of long-term BMI changes, rather than single BMI measures, on the risk of CVD and related outcomes. We particularly observed a strong significant gradient in heart failure risk which increased with more severe forms of obesity. This provides confirmatory evidence of the graded increase in heart failure risk with increasing obesity. The lack of a clear relationship between degree of obesity and the risk of peripheral vascular disease, as well as the reduced risk of peripheral vascular disease in the most severely obese group, is similar to findings in the Framingham heart study. As had been previously suggested [31], this unclear relationship may be either due to under-diagnosis of peripheral vascular disease, or a difference in the underlying disease mechanism compared to other types of CVD.

In relation solely to obesity, our findings in a large general adult population expand on those of a smaller study of 3070 Canadian adults which similarly found no significant change in individuals’ BMI over time [32]. Similarly, a retrospective cohort study of 11,735 adults with severe obesity (BMI 35 kg/m^2^ or greater) in the US, found that severely obese individuals remained in that BMI category over at least 5 years [7]. The current study is the largest prospective investigation to assess long term changes in BMI over time. Our finding that the general population of adults who were overweight or obese followed one of four stable upwards BMI trajectories over a decade, elaborates on previous research.

Previous studies of the association between obesity and mortality have produced conflicting results. In the original Framingham heart study and the Framingham offspring study, maximum BMI over a 24 year period was strongly associated with subsequent all-cause mortality [33]. However a systematic review of the risk of all-cause mortality in overweight and obese relative to normal weight individuals in the general population, found lower risk of mortality in overweight compared to normal weight subjects, but the highest mortality risk in more severely obese subjects with class 2 and 3 obesity [34]. More recently, a population-based cohort study found a J-shaped association between BMI and overall mortality such that lower BMI was associated with increased mortality risk, but the absolute mortality burden was predominantly driven by obesity [35]. In the current study, we observed a stepwise increase in the risk of all-cause and CVD-related mortality with increasing severity of obesity. This persisted after adjusting for the effect of age, sex, hypertension, type 2 diabetes, atrial fibrillation and chronic kidney disease. This observed association may be due to several plausible mechanisms. Severe obesity is a risk factor for dyslipidaemia and is associated with devastating health consequences such as obesity hypoventilation syndrome, obstructive sleep apnoea, liver disease and certain types of cancers [36], which could independently or synergistically increase the risk of mortality.

Some studies have reported an ‘Obesity paradox’ with clinically better outcomes in overweight and obese patients compared to normal weight patients in the context of prevalent cardiovascular disease such as heart failure [37] or following an acute coronary event [38]. In contrast, in the current study population, free from CVD at the start of follow-up, individuals with more severe obesity had earlier onset of incident CVD and earlier age at death, than overweight individuals. Our study provides compelling evidence of poor health outcomes associated with obesity.

## Conclusions

Despite widespread efforts to prevent and manage obesity, the majority of adults who are overweight or obese in the general population continue to remain so in the long term. This is associated with a three-fold increase in heart failure, cardiovascular disease and mortality risk in the stably severe obese population. This research highlights the high cardiovascular toll exacted by continuing failure to tackle obesity, particularly among more socio-economically deprived populations. More effective policies and weight-management interventions at societal, cultural and health service levels are needed to address this increasing burden. Further research is also needed to explore whether interventions to change BMI trajectories would have an impact on future CVD outcomes.

## Supplementary Information

**Additional file 1.** Supplementary online file showing cardiovascular disease diagnostic Read codes (list of diagnostic Read codes for coronary artery disease, peripheralvascular disease, cerebrovascular accident (Stroke) and transient ischaemic attack (TIA) and congestive cardiac failure (heart failure).

**Additional file 2: Table S1.** BIC for body mass index GBTM according to number of groups and trajectory shapes. **Table S2.** Average posterior probability and odds of correct classification for body mass index GBTM groups. **Table S3.** Sensitivity analyses of body mass index measures at baseline, 2, 5,8 and 10 years, by trajectory group (Analyses done using only BMI records available in GP records* (*n* = 260,962). **Table S4.** Risk of cardiovascular disease, heart failure and mortality in BMI trajectory groups 2, 3 and 4 compared to group 1. Sensitivity analyses restricted to individuals with CPRD data linked to hospital episode statistics and office of national statistics death records (*n* = 138,755). **Figure S1.** Sensitivity analyses of body mass index (BMI) trajectories using BMI measures at baseline and then follow-up at 2 years, 5 years, 8 years and 10 years (Analyses done using only records available in GP records (n = 260,962)).

## Data Availability

The CPRD and linked Hospital Episode Statistics datasets analysed during this study are available from the Clinical Practice Research Datalink (CPRD) (enquiries@cprd.com) but restrictions apply to the availability of these data, which were used under license for the current study, and so are not publicly available. Data are however available from the authors upon reasonable request and with permission of the CPRD Independent Scientific Advisory Committee (ISAC) (enquiries@cprd.com).

## Abbreviations

BMI: Body mass index
CHD: Coronary heart disease
CPRD: Clinical Practice Research datalink
CVD: Cardiovascular disease
GBTM: Group based trajectory modelling
HES: Hospital Episode Statistics
IMD: Index of Multiple Deprivation
ONS: Office for National Statistics
TIA: Transient ischaemic attack
