# Supplementary material for: Long-term body mass index changes in overweight and obese adults and the risk of heart failure, cardiovascular disease and mortality: a cohort study of over 260,000 adults in the UK
Source: BMC Public Health. 2021 Apr 15;21:576. doi: 10.1186/s12889-021-10606-1 (PMC8048253; doi:10.1186/s12889-021-10606-1)
Supplement: Supplementary file 1 — Additional file 1. Supplementary online file showing cardiovascular disease diagnostic Read codes (list of diagnostic Read codes for coronary artery disease, peripheralvascular disease, cerebrovascular accident (Stroke) and transient ischaemic attack (TIA) and congestive cardiac failure (heart failure). [file 12889_2021_10606_MOESM1_ESM.docx]

Online only – Supplementary file

Disease codes used for identification of cardiovascular disease (CVD) and heart failure

Cardiovascular disease diagnostic codes (Read code list)

**Coronary artery disease**

| Medical code | Read code | Read term |
| --- | --- | --- |
| 7783 | 323..00 | ECG: myocardial infarction |
| 26975 | 3233.00 | ECG: antero-septal infarct. |
| 26972 | 3234.00 | ECG:posterior/inferior infarct |
| 55401 | 3235.00 | ECG: subendocardial infarct |
| 52705 | 3236.00 | ECG: lateral infarction |
| 59032 | 323Z.00 | ECG: myocardial infarct NOS |
| 737 | 792..11 | Coronary artery bypass graft operations |
| 18249 | 7920.00 | Saphenous vein graft replacement of coronary artery |
| 11610 | 7920300 | Saphenous vein graft replacement of four+ coronary arteries |
| 7137 | 7920y00 | Saphenous vein graft replacement of coronary artery OS |
| 66236 | 7923200 | Prosthetic replacement of three coronary arteries |
| 5744 | 7927500 | Open angioplasty of coronary artery |
| 2901 | 7928.00 | Transluminal balloon angioplasty of coronary artery |
| 5703 | 7928.11 | Percutaneous balloon coronary angioplasty |
| 18670 | 7928000 | Percut transluminal balloon angioplasty one coronary artery |
| 42462 | 7928200 | Percut translum balloon angioplasty bypass graft coronary a |
| 41547 | 7928y00 | Transluminal balloon angioplasty of coronary artery OS |
| 732 | 7928z00 | Transluminal balloon angioplasty of coronary artery NOS |
| 33650 | 7929100 | Percut transluminal coronary thrombolysis with streptokinase |
| 8942 | 7929400 | Insertion of coronary artery stent |
| 42304 | 7929500 | Insertion of drug-eluting coronary artery stent |
| 6182 | 7929y00 | Other therapeutic transluminal op on coronary artery OS |
| 33471 | 792Dz00 | Other bypass of coronary artery NOS |
| 105184 | 792E.00 | Percutaneous coronary intervention |
| 107406 | 792E000 | Emergency percutaneous coronary intervention |
| 43939 | 793G.00 | Perc translumin balloon angioplasty stenting coronary artery |
| 61208 | 793Gz00 | Perc translum balloon angioplasty stenting coronary art NOS |
| 45960 | 8B27.00 | Antianginal therapy |
| 101121 | 8L40.00 | Coronary artery bypass graft operation planned |
| 101373 | 8L41.00 | Coronary angioplasty planned |
| 240 | G3...00 | Ischaemic heart disease |
| 24783 | G3...11 | Arteriosclerotic heart disease |
| 20416 | G3...12 | Atherosclerotic heart disease |
| 1792 | G3...13 | IHD - Ischaemic heart disease |
| 241 | G30..00 | Acute myocardial infarction |
| 2491 | G30..12 | Coronary thrombosis |
| 30421 | G30..13 | Cardiac rupture following myocardial infarction (MI) |
| 1677 | G30..15 | MI - acute myocardial infarction |
| 13571 | G30..16 | Thrombosis - coronary |
| 17689 | G30..17 | Silent myocardial infarction |
| 12139 | G300.00 | Acute anterolateral infarction |
| 5387 | G301.00 | Other specified anterior myocardial infarction |
| 40429 | G301000 | Acute anteroapical infarction |
| 17872 | G301100 | Acute anteroseptal infarction |
| 14897 | G301z00 | Anterior myocardial infarction NOS |
| 8935 | G302.00 | Acute inferolateral infarction |
| 29643 | G303.00 | Acute inferoposterior infarction |
| 23892 | G304.00 | Posterior myocardial infarction NOS |
| 14898 | G305.00 | Lateral myocardial infarction NOS |
| 63467 | G306.00 | True posterior myocardial infarction |
| 3704 | G307.00 | Acute subendocardial infarction |
| 9507 | G307000 | Acute non-Q wave infarction |
| 10562 | G307100 | Acute non-ST segment elevation myocardial infarction |
| 1678 | G308.00 | Inferior myocardial infarction NOS |
| 30330 | G309.00 | Acute Q-wave infarct |
| 32854 | G30B.00 | Acute posterolateral myocardial infarction |
| 29758 | G30X.00 | Acute transmural myocardial infarction of unspecif site |
| 12229 | G30X000 | Acute ST segment elevation myocardial infarction |
| 34803 | G30y.00 | Other acute myocardial infarction |
| 28736 | G30y000 | Acute atrial infarction |
| 62626 | G30y100 | Acute papillary muscle infarction |
| 41221 | G30y200 | Acute septal infarction |
| 46017 | G30yz00 | Other acute myocardial infarction NOS |
| 14658 | G30z.00 | Acute myocardial infarction NOS |
| 27951 | G31..00 | Other acute and subacute ischaemic heart disease |
| 23579 | G310.00 | Postmyocardial infarction syndrome |
| 15661 | G310.11 | Dressler's syndrome |
| 36523 | G311.00 | Preinfarction syndrome |
| 4656 | G311.11 | Crescendo angina |
| 1431 | G311.13 | Unstable angina |
| 19655 | G311.14 | Angina at rest |
| 61072 | G311000 | Myocardial infarction aborted |
| 55137 | G311011 | MI - myocardial infarction aborted |
| 7347 | G311100 | Unstable angina |
| 17307 | G311200 | Angina at rest |
| 34328 | G311300 | Refractory angina |
| 18118 | G311400 | Worsening angina |
| 11983 | G311500 | Acute coronary syndrome |
| 54251 | G311z00 | Preinfarction syndrome NOS |
| 39449 | G312.00 | Coronary thrombosis not resulting in myocardial infarction |
| 9413 | G31y.00 | Other acute and subacute ischaemic heart disease |
| 9276 | G31y000 | Acute coronary insufficiency |
| 68357 | G31y100 | Microinfarction of heart |
| 27977 | G31yz00 | Other acute and subacute ischaemic heart disease NOS |
| 4017 | G32..00 | Old myocardial infarction |
| 1430 | G33..00 | Angina pectoris |
| 20095 | G330.00 | Angina decubitus |
| 18125 | G330000 | Nocturnal angina |
| 29902 | G330z00 | Angina decubitus NOS |
| 11048 | G331.11 | Variant angina pectoris |
| 36854 | G332.00 | Coronary artery spasm |
| 25842 | G33z.00 | Angina pectoris NOS |
| 1414 | G33z300 | Angina on effort |
| 9555 | G33z500 | Post infarct angina |
| 26863 | G33z600 | New onset angina |
| 12804 | G33z700 | Stable angina |
| 28554 | G33zz00 | Angina pectoris NOS |
| 28138 | G34..00 | Other chronic ischaemic heart disease |
| 5413 | G340.00 | Coronary atherosclerosis |
| 1344 | G340.12 | Coronary artery disease |
| 3999 | G340000 | Single coronary vessel disease |
| 5254 | G340100 | Double coronary vessel disease |
| 29421 | G344.00 | Silent myocardial ischaemia |
| 34633 | G34y.00 | Other specified chronic ischaemic heart disease |
| 24540 | G34y000 | Chronic coronary insufficiency |
| 23078 | G34y100 | Chronic myocardial ischaemia |
| 35713 | G34yz00 | Other specified chronic ischaemic heart disease NOS |
| 15754 | G34z.00 | Other chronic ischaemic heart disease NOS |
| 18889 | G34z000 | Asymptomatic coronary heart disease |
| 18842 | G35..00 | Subsequent myocardial infarction |
| 45809 | G350.00 | Subsequent myocardial infarction of anterior wall |
| 38609 | G351.00 | Subsequent myocardial infarction of inferior wall |
| 72562 | G353.00 | Subsequent myocardial infarction of other sites |
| 46166 | G35X.00 | Subsequent myocardial infarction of unspecified site |
| 36423 | G36..00 | Certain current complication follow acute myocardial infarct |
| 24126 | G360.00 | Haemopericardium/current comp folow acut myocard infarct |
| 23708 | G361.00 | Atrial septal defect/curr comp folow acut myocardal infarct |
| 37657 | G362.00 | Ventric septal defect/curr comp fol acut myocardal infarctn |
| 59940 | G364.00 | Ruptur chordae tendinae/curr comp fol acute myocard infarct |
| 69474 | G365.00 | Rupture papillary muscle/curr comp fol acute myocard infarct |
| 32272 | G38..00 | Postoperative myocardial infarction |
| 46112 | G380.00 | Postoperative transmural myocardial infarction anterior wall |
| 46276 | G381.00 | Postoperative transmural myocardial infarction inferior wall |
| 106812 | G383.00 | Postoperative transmural myocardial infarction unspec site |
| 41835 | G384.00 | Postoperative subendocardial myocardial infarction |
| 68748 | G38z.00 | Postoperative myocardial infarction, unspecified |
| 22383 | G3y..00 | Other specified ischaemic heart disease |
| 1676 | G3z..00 | Ischaemic heart disease NOS |
| 35119 | G501.00 | Post infarction pericarditis |
| 52517 | Gyu3.00 | [X]Ischaemic heart diseases |
| 39546 | Gyu3000 | [X]Other forms of angina pectoris |
| 68401 | Gyu3200 | [X]Other forms of acute ischaemic heart disease |
| 47637 | Gyu3300 | [X]Other forms of chronic ischaemic heart disease |
| 96838 | Gyu3400 | [X]Acute transmural myocardial infarction of unspecif site |
| 109035 | Gyu3500 | [X]Subsequent myocardial infarction of other sites |
| 99991 | Gyu3600 | [X]Subsequent myocardial infarction of unspecified site |
| 40887 | N23yB00 | Ischaemic infarction of muscle |

**Peripheral vascular disease**

| Medical code | Read code | Read term |
| --- | --- | --- |
| 5943 | G73..00 | Other peripheral vascular disease |
| 5702 | G73..11 | Peripheral ischaemic vascular disease |
| 6827 | G73..13 | Peripheral ischaemia |
| 9204 | G732.00 | Peripheral gangrene |
| 105317 | G734.00 | Peripheral arterial disease |
| 38907 | G73y.00 | Other specified peripheral vascular disease |
| 4325 | G73yz00 | Other specified peripheral vascular disease NOS |
| 3530 | G73z.00 | Peripheral vascular disease NOS |
| 1517 | G73z000 | Intermittent claudication |
| 101866 | G73z012 | Vascular claudication |
| 2760 | G73zz00 | Peripheral vascular disease NOS |
| 15302 | G742z00 | Peripheral arterial embolism and thrombosis NOS |
| 73961 | Gyu7400 | [X]Other specified peripheral vascular diseases |

**Cerebrovascular accident (Stroke) and Transient ischaemic attack (TIA)**

| Medical code | Read code | Read term |
| --- | --- | --- |
| 569 | G64..12 | Infarction - cerebral |
| 1469 | G66..00 | Stroke and cerebrovascular accident unspecified |
| 1895 | G65z.00 | Transient cerebral ischaemia NOS |
| 2418 | G6...00 | Cerebrovascular disease |
| 3149 | G64z.00 | Cerebral infarction NOS |
| 5184 | G670.11 | Precerebral atherosclerosis |
| 5363 | G64..11 | CVA - cerebral artery occlusion |
| 5602 | G64z.12 | Cerebellar infarction |
| 6116 | G66..13 | CVA - Cerebrovascular accident unspecified |
| 6155 | G64..13 | Stroke due to cerebral arterial occlusion |
| 6228 | G68X.00 | Sequelae of stroke,not specfd as h'morrhage or infarction |
| 6253 | G66..12 | Stroke unspecified |
| 6960 | G61..11 | CVA - cerebrovascular accid due to intracerebral haemorrhage |
| 8443 | G663.00 | Brain stem stroke syndrome |
| 8837 | G64..00 | Cerebral arterial occlusion |
| 9985 | G64z200 | Left sided cerebral infarction |
| 10062 | G6z..00 | Cerebrovascular disease NOS |
| 10504 | G64z300 | Right sided cerebral infarction |
| 11171 | G670.00 | Cerebral atherosclerosis |
| 13577 | G67..00 | Other cerebrovascular disease |
| 15019 | G641.00 | Cerebral embolism |
| 15788 | G65zz00 | Transient cerebral ischaemia NOS |
| 16517 | G640.00 | Cerebral thrombosis |
| 17322 | G664.00 | Cerebellar stroke syndrome |
| 18604 | G61..12 | Stroke due to intracerebral haemorrhage |
| 19354 | G65y.00 | Other transient cerebral ischaemia |
| 23671 | G63y000 | Cerebral infarct due to thrombosis of precerebral arteries |
| 24446 | G63y100 | Cerebral infarction due to embolism of precerebral arteries |
| 27975 | G641000 | Cerebral infarction due to embolism of cerebral arteries |
| 33543 | G6X..00 | Cerebrl infarctn due/unspcf occlusn or sten/cerebrl artrs |
| 34117 | G67y.00 | Other cerebrovascular disease OS |
| 36717 | G640000 | Cerebral infarction due to thrombosis of cerebral arteries |
| 37493 | G67z.00 | Other cerebrovascular disease NOS |
| 40053 | G671.00 | Generalised ischaemic cerebrovascular disease NOS |
| 40758 | G6W..00 | Cereb infarct due unsp occlus/stenos precerebr arteries |
| 45781 | G63..00 | Precerebral arterial occlusion |
| 51138 | G68W.00 | Sequelae/other + unspecified cerebrovascular diseases |
| 51311 | G6y..00 | Other specified cerebrovascular disease |
| 51326 | G63y.00 | Other precerebral artery occlusion |
| 51759 | G677000 | Occlusion and stenosis of middle cerebral artery |
| 53745 | Gyu6400 | [X]Other cerebral infarction |
| 57495 | G63..11 | Infarction - precerebral |
| 57527 | G677100 | Occlusion and stenosis of anterior cerebral artery |
| 63746 | Fyu5500 | [X]Other transnt cerebral ischaemic attacks+related syndroms |
| 65770 | G677200 | Occlusion and stenosis of posterior cerebral artery |
| 70536 | G671000 | Acute cerebrovascular insufficiency NOS |
| 71274 | G677400 | Occlusion+stenosis of multiple and bilat cerebral arteries |
| 71585 | G63z.00 | Precerebral artery occlusion NOS |
| 73901 | Gyu6.00 | [X]Cerebrovascular diseases |
| 91627 | Gyu6300 | [X]Cerebrl infarctn due/unspcf occlusn or sten/cerebrl artrs |
| 92036 | Gyu6600 | [X]Occlusion and stenosis of other cerebral arteries |
| 94482 | Gyu6G00 | [X]Cereb infarct due unsp occlus/stenos precerebr arteries |
| 98188 | G679.00 | Small vessel cerebrovascular disease |
| 98642 | G633.00 | Multiple and bilateral precerebral arterial occlusion |
| 110337 | Gyu6C00 | [X]Sequelae of stroke,not specfd as h'morrhage or infarction |
| 111096 | Gyu6700 | [X]Other specified cerebrovascular diseases |

**Congestive cardiac failure (Heart failure)**

| Medical code | Read code | Read term |
| --- | --- | --- |
| 23707 | G580000 | Acute congestive heart failure |
| 27964 | G582.00 | Acute heart failure |
| 5255 | G581000 | Acute left ventricular failure |
| 23481 | G581.11 | Asthma - cardiac |
| 9524 | G580.14 | Biventricular failure |
| 1223 | G58..11 | Cardiac failure |
| 17278 | G58z.12 | Cardiac failure NOS |
| 32671 | G580100 | Chronic congestive heart failure |
| 11424 | G580300 | Compensated cardiac failure |
| 2906 | G580.11 | Congestive cardiac failure |
| 398 | G580.00 | Congestive heart failure |
| 94870 | G580400 | Congestive heart failure due to valvular disease |
| 27884 | G580200 | Decompensated cardiac failure |
| 2062 | G58..00 | Heart failure |
| 4024 | G58z.00 | Heart failure NOS |
| 5942 | G581.13 | Impaired left ventricular function |
| 12550 | G5yyA00 | Left ventricular diastolic dysfunction |
| 884 | G581.00 | Left ventricular failure |
| 8966 | G5yy900 | Left ventricular systolic dysfunction |
| 18853 | 662f.00 | New York Heart Association classification - class I |
| 13189 | 662g.00 | New York Heart Association classification - class II |
| 19066 | 662h.00 | New York Heart Association classification - class III |
| 51214 | 662i.00 | New York Heart Association classification - class IV |
| 43618 | G581.12 | Pulmonary oedema - acute |
| 22262 | G1yz100 | Rheumatic left ventricular failure |
| 10079 | G580.12 | Right heart failure |
| 10154 | G580.13 | Right ventricular failure |
| 12590 | G58z.11 | Weak heart |
