# Supplementary material for: Long-term body mass index changes in overweight and obese adults and the risk of heart failure, cardiovascular disease and mortality: a cohort study of over 260,000 adults in the UK
Source: BMC Public Health. 2021 Apr 15;21:576. doi: 10.1186/s12889-021-10606-1 (PMC8048253; doi:10.1186/s12889-021-10606-1)
Supplement: Supplementary file 2 — Additional file 2: Table S1. BIC for body mass index GBTM according to number of groups and trajectory shapes. Table S2. Average posterior probability and odds of correct classification for body mass index GBTM groups. Table S3. Sensitivity analyses of body mass index measures at baseline, 2, 5,8 and 10 years, by trajectory group (Analyses done using only BMI records available in GP records* (n = 260,962). Table S4. Risk of cardiovascular disease, heart failure and mortality in BMI trajectory groups 2, 3 and 4 compared to group 1. Sensitivity analyses restricted to individuals with CPRD data linked to hospital episode statistics and office of national statistics death records (n = 138,755). Figure S1. Sensitivity analyses of body mass index (BMI) trajectories using BMI measures at baseline and then follow-up at 2 years, 5 years, 8 years and 10 years (Analyses done using only records available in GP records (n = 260,962)). [file 12889_2021_10606_MOESM2_ESM.docx]

**Supplementary tables**

**Supplementary Table 1. BIC for body mass index GBTM according to number of groups and trajectory shapes.**

| Number of groups | Trajectory shapes ^1^ | BIC ^2^ (n=264,230) |
| --- | --- | --- |
| 2 | 0 0 | -3707758.71 |
| 3 | 0 0 0 | -3510621.69 |
| 4 | 0 0 0 0 | -3395075.03 |
| 4 | 0 0 0 1 | -3394240.84 |
| 4 | 0 0 1 1 | -3389806.86 |
| 4 | 0 1 1 1 | -3380072.27 |
| 4 | 1 1 1 1 | -3373466.80 |
| 4 | 1 1 1 2 | -3373381.52 |
| 4 | 1 1 2 2 | -3373372.22 |
| 4 | 1 2 2 2 | -3373349.12 |
| 4 | 2 2 2 2 | -3373287.49 |
| 4 | 2 2 2 3 | -3373141.88 |
| 4 | 2 2 3 3 | -3372451.87 |
| 4 | 2 3 3 3 | -3371973.99 |
| 4 | 3 3 3 3 | **-3371582.03** |
| 4 | 1 2 3 3 | -3372515.26 |
| 4 | 3 3 3 2 | -3371722.52 |
| 5 | 0 0 0 0 0 * | -3323263.68 |
| 6 | 0 0 0 0 0 0 * | -3276741.64 |
| 7 | 0 0 0 0 0 0 0 * | -3246740.47 |
| 8 | 0 0 0 0 0 0 0 0 * | -3227500.80 |

^1^ Trajectory shapes: 0 = zero-order; 1=linear; 2=quadratic; 3=cubic

^2^ BIC – Bayesian Information Criterion (for the total number of participants)

* One or more of the groups had a small proportion of observations

**Supplementary Table 2. Average posterior probability and odds of correct classification for body mass index GBTM groups.**

|  | **Body mass index trajectory groups** | | | |
| --- | --- | --- | --- | --- |
|  | **1** | **2** | **3** | **4** |
| Count per group | 95,944 | 104,616 | 50,866 | 12,804 |
| **Average posterior probability** | 0.95 | 0.93 | 0.95 | 0.97 |
| **Odds of correct classification** | 32.3 | 20.3 | 80.1 | 736.4 |

**Supplementary table 3. Body mass index measures at baseline, 2, 5,8 and 10 years, by trajectory group (Analyses done using only BMI records available in GP records* (n=260,962)**

| **Body mass index in kg/m^2^** | Trajectory 1  (n=107,134) | Trajectory 2  (n=100,863) | Trajectory 3  (n=43,019) | Trajectory 4  (n=9,946) |
| --- | --- | --- | --- | --- |
| Baseline BMI (mean(SD)) | n=107,134  28.95 (2.32) | n=100,863  34.36 (2.75) | n=43,019  40.94 (3.51) | n=9,946  50.29 (5.78) |
| BMI at 2 years (mean(SD)) | n=78,457  28.91 (2.43) | n=79,356  34.44 (2.62) | n=36,130  40.97 (3.35) | n=8,645  50.34 (5.60) |
| BMI at 5 years (mean(SD)) | n=51,434  29.40 (2.64) | n=51,048  35.04 (2.85) | n=22,668  41.73 (3.67) | n=5,170  51.08 (5.90) |
| BMI at 8 years (mean(SD)) | n=43,465  29.60 (2.82) | n=41,638  35.43 (3.07) | n=18,007  42.20 (3.93) | n=3,876  51.60 (6.00) |
| BMI at 10 years (mean(SD)) | n=49,994  29.77 (3.04) | n=45,033  35.70 (3.39) | n=18,711  42.43 (4.39) | n=3,721  51.62 (6.6) |
| Mean BMI change over 10 years | 0.83 (3.48) | 1.48 (4.40) | 1.71 (5.68) | 2.03 (8.07) |

*Analyses excludes individuals who had only one BMI before the incidence of CVD, heart failure or mortality

**Supplementary table 4. Risk of cardiovascular disease, heart failure and mortality in BMI trajectory groups 2, 3 and 4 compared to group 1. Sensitivity analyses restricted to individuals with CPRD data linked to hospital episode statistics and office of national statistics death records (n=138,755)**

|  |  | Unadjusted Hazards ratio (95% CI) | Adjusted hazards ratio (95% CI) | |
| --- | --- | --- | --- | --- |
| CVD outcome | Trajectory group |  | Model adjusted for age and sex | Model adjusted for age, sex and comorbidities** |
| Overall CVD | overweight-S  obese 1-S  obese 2-S  obese 3-S | 1.00  1.31 (1.27-1.36)  1.39 (1.33-1.45)  1.60 (1.50-1.72) | 1.00  1.25 (1.21-1.29)  1.47 (1.41-1.53)  1.85 (1.72-1.97) | 1.00  1.17 (1.13-1.22)  1.29 (1.24-1.35)  1.51 (1.41-1.61) |
| Coronary heart disease | overweight-S  obese 1-S  obese 2-S  obese 3-S | 1.00  1.28 (1.23-1.34)  1.25 (1.19-1.32)  1.21 (1.10-1.34) | 1.00  1.23 (1.18-1.29)  1.34 (1.27-1.41)  1.41 (1.28-1.56) | 1.00  1.16 (1.11-1.21)  1.18 (1.12-1.25)  1.16 (1.05-1.28) |
| Stroke/ TIA | overweight-S  obese 1-S  obese 2-S  obese 3-S | 1.00  1.26 (1.18-1.34)  1.19 (1.09-1.28)  1.14 (0.98-1.32) | 1.00  1.18 (1.11-1.26)  1.22 (1.13-1.33)  1.26 (1.09--1.46) | 1.00  1.12 (1.05-1.19)  1.10 (1.01-1.19)  1.05 (0.91-1.22) |
| Peripheral vascular disease | overweight-S  obese 1-S  obese 2-S  obese 3-S | 1.00  1.16 (1.05-1.29)  1.06 (0.93-1.20)  0.91 (0.71-1.16) | 1.00  1.11 (1.00-1.23)  1.15 (1.02-1.31)  1.12 (0.88-1.42) | 1.00  0.89 (0.92-1.09)  0.91 (0.80-1.03)  0.78 (0.61-1.00) |
| Heart failure | overweight-S  obese 1-S  obese 2-S  obese 3-S | 1.00  1.85 (1.71-2.00)  2.49 (2.28-2.71)  4.32 (3.86-4.84) | 1.00  1.73 (1.60-1.87)  2.71 (2.48-2.95)  5.39 (4.81-6.05) | 1.00  1.54 (1.42-1.66)  2.11 (1.93-2.31)  3.60 (3.21-4.05) |
| All-cause mortality | overweight-S  obese 1-S  obese 2-S  obese 3-S | 1.00  1.29 (1.24-1.34)  1.60 (1.52-1.67)  2.62 (2.45-2.79) | 1.00  1.20 (1.15-1.25)  1.71 (1.63-1.79)  3.18 (2.98-3.40) | 1.00  1.17 (1.13-1.22)  1.63 (1.56-1.71)  2.94 (2.75-3.14) |
| CVD-related deaths | overweight-S  obese 1-S  obese 2-S  obese 3-S | 1.00  1.47 (1.34-1.61)  1.82 (1.64-2.02)  2.65 (2.28-3.07) | 1.00  1.37 (1.25-1.51)  2.03 (1.83-2.25)  3.49 (3.00-4.06) | 1.00  1.29 (1.18-1.42)  1.80 (1.62-2.00)  2.89 (2.48-3.36) |

Trajectory group 1 (overweight-stable group) used as baseline group for comparison

**Multivariate cox regression models adjusted for age, sex, hypertension, type 2 diabetes, atrial fibrillation and chronic kidney disease

**Supplementary figures**

**Supplementary Figure 1 Body mass index (BMI) trajectories using BMI measures at baseline and then follow-up at 2 years, 5 years, 8 years and 10 years (Analyses done using only records available in GP records (n=260,962))**


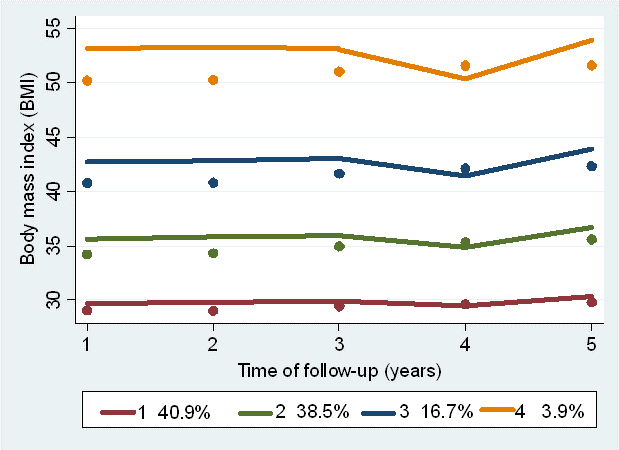


Percentages below plot represent percentage of study population within each trajectory group

Mean BMI change in trajectory group 1 (overweight-stable group): +0.83 (SD 3.48) kg/m^2^

Mean BMI change in trajectory group 2 (obese class 1-stable group): +1.48 (SD 4.40) kg/m^2^

Mean BMI change in trajectory group 3 (obese class 2-stable group): +1.71 (SD 5.68) kg/m^2^

Mean BMI change in trajectory group 4 (obese class 3-stable group): +2.03 (SD 8.07) kg/m^2^
